# Adaptation and Latent Structure of the Swahili Version of Beck Depression Inventory-II in a Low Literacy Population in the Context of HIV

**DOI:** 10.1371/journal.pone.0151030

**Published:** 2016-06-03

**Authors:** Amina Abubakar, Raphael Birya Kalu, Khamis Katana, Beatrice Kabunda, Amin S. Hassan, Charles R. Newton, Fons Van de Vijver

**Affiliations:** 1 KEMRI/Wellcome Trust Research Programme, Centre for Geographic Medicine Research (Coast), Kilifi, Kenya; 2 Tilburg University, Tilburg, the Netherlands; 3 Utrecht University, Utrecht, the Netherlands; 4 Neurosciences Unit, Institute of Child Health, University College London, London, United Kingdom; 5 London School of Hygiene and Tropical Medicine, London, United Kingdom; 6 Department of Psychiatry, University of Oxford, Oxford, United Kingdom; 7 Centre for International Health and Development, Institute of Child Health, London, United Kingdom; 8 North-West University, Potchefstroom, South Africa; 9 University of Queensland, Brisbane, Australia; Federal University of Rio de Janeiro, BRAZIL

## Abstract

**Objective:**

We set out to adapt the Beck Depression Inventory (BDI)-II in Kenya and examine its factorial structure.

**Methods:**

In the first phase we carried out in-depth interviews involving 29 adult members of the community to elicit their understanding of depression and identify aspects of the BDI-II that required adaptation. In the second phase, a modified version of BDI-II was administered to 221 adults randomly selected from the community to allow for the evaluation of its psychometric properties. In the third phase of the study we evaluated the discriminative validity of BDI-11 by comparing a randomly chosen community sample (n = 29) with caregivers of adolescents affected by HIV (n = 77).

**Results:**

A considerable overlap between the BDI symptoms and those generated in the interviews was observed. Relevant idioms and symptoms such as ‘thinking too much’ and ‘*Kuchoka moyo* (having a tired heart)’ were identified. The administration of the BDI had to be modified to make it suitable for the low literacy levels of our participants. Fit indices for several models (one factorial, two-factor model and a three factor model) were all within acceptable range. Evidence indicated that while multidimensional models could be fitted, the strong correlations between the factors implied that a single factor model may be the best suited solution (alpha [0.89], and a significant correlation with locally identified items [*r* = 0.51]) confirmed the good psychometric properties of the adapted BDI-II. No evidence was found to support the hypothesis that somatization was more prevalent. Lastly, caregivers of HIV affected adolescents had significantly higher scores compared to adults randomly selected from the community *F*(1, 121) = 23.31, *p* < .001 indicating the discriminative validity of the adapted BDI = II.

**Conclusions:**

With an adapted administration procedure, the BDI-II provides an adequate measure of depressive symptoms which can be used alongside other measures for proper diagnosis in a low literacy population.

## Introduction

Recent estimates indicate that depression is highly prevalent in many developing countries [1,2]; yet there is a shortage of adequately standardized measures to identify and diagnose those in need of mental health services [3]. In Western countries there are many standardized and widely used measures to assess depressive symptoms both for clinical and research purpose. One such measure is the Beck Depression Inventory (BDI-II; [4]), which has been widely used across many cultural contexts [5]. The BDI-II is a self-report measure that was constructed to assess depressive symptoms as per DSM-IV criteria. The use of well-validated measures such as BDI-II may potentially provide a quick solution to a shortage of measures in non-Western settings. However, the transfer of mental health measures from one context to another can be accompanied by significant limitations of interpretation.

Problems of transfer of mental health measures can arise from three sources of bias [6]. *Construct bias* occurs when an instrument only partially samples the domains that define a construct or includes items that are culturally inappropriate or irrelevant to the construct being studied. For instance, in developing the Shona Symptoms Questionnaire it was observed that the syndrome closest to depression among the Shona of Zimbabwe could not be adequately measured by the World Health Organization (WHO) depressive symptoms measures because there were various symptoms that were not captured in the WHO measures [7]. *Method bias* refers to problems due to instrument characteristics and test administration procedures. Included here are inaccuracies in results arising from comparing two samples that differ in different test taking behaviour [8]. For instance in contexts where people are highly literate, self-administration is the common way of delivery of mental health measures. Therefore a scale such as the BDI-II uses the first person phrasing which may create confusion when administering it as an interviewer assisted scale (necessary when participants are not literate). The last form of bias concerns *item bias*, which occurs when items have a different meaning or difficulty in different communities. This differential meaning may arise from poor translations of items [6] or lack of semantic equivalence of original and translated items. For instance, in a study to validate the Edinburgh Postnatal Depression Scale (EPDS; [9]) in Ethiopia it was observed that the Amharic term for ‘restless’ was often misunderstood by participants who would respond, ‘no, I was not restless. I had no chores to do, so I was resting most of the day’ (*pg* 104)’ [10]. This response implies that despite the semantic (linguistic) equivalence the term did not have conceptual equivalence.

Given these concerns of using tests in various contexts there is a need to ensure that the tests are validated and properly adapted to the new context. The current study set out to adapt the BDI-II for people who are not literate. Beyond adapting the BDI-II [4] to the new cultural setting, there are other psychometric issues surrounding the measure that we aimed to address in this study. Despite its popularity, the factorial structure of the BDI remains controversial. Various investigators report 1, 2 or 3 factors models variously labelled as, among others, somatic, cognitive, and cognitive affective (for details, see Quilty *et al*. [11]).

An important issue of both theoretical and clinical significance in the evaluation of depressive symptoms in Africa is somatisation. It has been previously suggested in the literature that in many non-Western settings, especially in Africa and Asia, there was a higher tendency for patients with depression to present with somatic symptoms as opposed to psychological symptoms. The direct implication of this is that Western based measures with their emphasis on psychological symptoms may not be able to adequately evaluate depressive symptoms in a non-Western setting.

The main impetus for adapting mental health questionnaires for use in our context is the need to have adequate measures for evaluating the impact of various health conditions on human functioning. In our case we were interested in getting a measure that could be used to describe mental health, especially depressive symptoms, in the context of HIV/AIDS. Sub-Saharan Africa is home to 68% of all HIV-infected adults, 90% of all HIV-infected children. Millions of families are affected by HIV in various ways including caring for someone with HIV, taking over the care of children who have been orphaned by HIV or living with someone who is experiencing an HIV related illness. There is evidence to indicate that adult caregivers of children affected by HIV experience significant caregiving stress and present with more mental health compared to community controls [12–14].

Taking this into consideration, we set out to adapt the BDI-II by dealing with sources of bias mentioned above [15] and to use Confirmatory Factor Analysis (CFA) to evaluate the factorial structure. In addition, we also we set out to evaluate if the adapted Swahili BDI would be sensitive to mental health problems in HIV-affected families. Using a three phased study design we set out to:

1. Highlight modifications made to the BDI-II through a systematic adaptation process
2. Evaluate the latent structure of the BDI-II to determine the best fitting model and how best to score BDI-II in this setting.
3. Examine the extent to which people in our context are more likely to report somatic symptoms compared to the other symptoms.
4. Evaluate the discriminant validity and usefulness of the BDI in examining depressive symptoms among adults in the context of HIV infection.

## Methods

### Study site

The study was undertaken in Kilifi County, on the Kenyan Coast. The study took place within a demarcated area of the county that undergoes active, four-monthly demographic surveillance, in which the births, deaths, and movements of individuals are recorded at the Centre for Geographic Medicine Research (Coast)—KEMRI. An approximated 71% of the county population live below the poverty line [a monthly income of less than $18 and $33 for rural and urban dwellers, respectively] [16]. A significant number of adults have limited if any education and are unable to adequately participate in written interviews. The majority of the population in Kilifi belong to the Mijikenda ethnic group. Two Bantu languages are commonly spoken in the area, Kigiryama (the local vernacular) and Swahili (the lingua franca and the national language). Within Kilifi County Hospital, there is one specialist psychiatric clinic, which is run by a trained nurse and serves up to 70 patients a day. The most common problem presented is schizophrenia followed by depression and other mood disorders (Mr Nassor, personal communication). There were no formal measures for evaluation and diagnosis of depression at the time of this study.

### Ethics statement

The Kenya Medical Research Institute National Scientific and Ethical Committees approved the study. Written informed consent was obtained from all study participants prior to participation.

### Study design

The study was carried out in three phases. In the first phase we carried out a largely qualitative study among community members and health professionals so as to understand local terms and idioms used to express depressive symptoms and to gain information to adequately adapt the English version of BDI-11 into the Swahili language. In the second phase, we administered the adapted Swahili version to a sample of 221 participants and used the data to evaluate the psychometric characteristics of BDI. Lastly, 106 adults were administered to the BDI-11 in the third phase of the study so as to evaluate the discriminant validity of BDI-11. In the following sections of the manuscripts we will present the aims, methodology and results of each of these phases before presenting a general discussion of our findings.

### Phase one

#### Aim

To translate the BDI-II into Swahili, make adaptations to the BDI-II so as to ensure it is appropriate to examine both the cultural context and sample characteristics. We chose to adapt the BDI-II into Swahili because almost everyone within the study areas understands the language, which is the most widely spoken language in East Africa.

#### Sample

A random sample of 29 people from the community took part in this study. Sample selection was carried out so as to include both literate and non-literate individuals, health professional (e.g. nurses and doctors), teachers, farmers, fisher-folk, amongst others, with the requirement that participants were familiar with either of the local languages (Swahili and Kigiryama) and also familiar with the Giryama culture.

#### Method

In-depth interviews (n = 29) were carried out with structured open-ended guidelines. First the interviewer presented a vignette to elicit the term for depression. A vignette based on depressive symptoms following a stressful event was used since it was relatively easy for our participants to relate to this. The description given to the participants was: *‘As part of life*, *people do meet various challenges and difficult circumstances*, *e*.*g*. *loss of a loved one*. *Sometimes such circumstances affect us leading to adverse outcomes (hurts a lot leading to specific symptoms*, *showing we are not coping well) have you ever been in this kind of situation*? *Do you know anyone who has experienced this*? *What Swahili or Kigiryama word would you use to describe this*? *What are the symptoms one would elicit in this condition*? *[We provide the best possible translation into English]*. In this phase of the study we also interviewed clinicians and nurses who were familiar with the local community and culture.

For some of the participants (n = 18), following this first task we then administered the fully translated version of the BDI-II. After the administration of the BDI-II questions participants were requested to evaluate each item on relevancy, clarity of item wording, and appropriateness for the context. The participants were also requested to make suggestions on extra items that they thought were relevant to the community but had not been discussed. Notes were taken during the interviews. Written notes were content analysed independently by different investigators to identify recurrent themes.

#### Results

Early in the adaptation process the BDI format for administering the questionnaire was found to be inappropriate. Those interviewed found it hard to remember all the response options (ranging from 4 to 7 categories), which led to the interviews taking longer since the interviewer had to repeat the options several times.

With the exception of one clinician, none of the respondents interviewed were aware of any direct term or word, either in Swahili or Kigiryama, that adequately explained the whole set of symptoms or syndrome; but gave suggestions of possible terms such as *‘simanzi’* (deep sadness or sorrow), *kugandamizwa*, *kutsanganyikiriwa* (literally meaning getting mixed up or confused). One clinician pointed out that there is a term for depression in Swahili (*Unyogovu)*, but this term is not used by people in everyday discourse; hence, it is not a useful term from a day-to-day practical perspective.

Following our content analysis of the interview notes we made a list of symptoms closely associated with depressive moods in the study area (see S1 Table for the list). An evaluation of the list indicated that there was a significant overlap between what was mentioned by the community and what is assessed by the BDI-II. There were a few new items identified by the community, which included; thinking a lot, poor hygienic standards, negligence of work responsibility, and poor performance at work.

In rating each of the BDI-II items community members suggested ways in which items could be reworded to avoid ambiguity in wording, yet none of the items were deemed to be inappropriate or irrelevant to the construct of the instrument. Initial reservations from the study team on the cultural appropriateness of item 21 ‘lost interest in sex’ were unfounded, when the interviewees indicated that though embarrassing and hardly discussed within their cultural context, loss of interest in sexual relationship is an important symptom of distress and mental health in general. Only one participant thought that the question was too private and should not be asked.

#### Modifications and Conclusions

Based on the above information, various modifications were made to the Swahili version of the BDI-II:

- Given the general similarity between the symptoms in the BDI-II and items mentioned during the interviews and since none of the items were specifically picked out for exclusion from the questionnaire, all the 21 items were retained.
- The administration procedures were changed. The most significant change made during this process involved the change of administration mode to a conversational manner. Each of the BDI statements had an introductory statement that allowed the interview to be carried out in a more relaxed and conversational approach aimed to make the interview process flow in a natural manner. We first asked the participants if they had experienced any changes in a specific item in the last 2 weeks (Have you experienced any changes in your appetite in the last 2 weeks?). If they said no, then we would score the lowest (zero). If they said yes we would ask them what changes (So what kind of change did you experience; for example did you eat more or did you eat less?). When they said they ate a lot, we would then present one of the two response options and ask them to pick which one describes their condition best.
- From the list of items identified from the community, we consulted with a psychiatric nurse and based on the discussion we chose three items to be included in the questionnaire to provide extra validity. The main selection criteria was the extent to which the psychiatric nurse thought the items were salient in our context based on their long clinical experience. These items are: (1) In the past two weeks have you been thinking too much? (2) Have you been unable to carry out your work duties? (3) Have you seen a drop in your ability to take proper care of yourself? Answer options were *yes* and *no*. Moreover, we added a single open-ended question for participants to indicate if they have been experiencing any other symptoms/concerns not previously mentioned.
- The final items were checked by a p*anel* of six professionals; three fieldworkers, a psychologist, epidemiologist and a linguist were asked to evaluate the conceptual equivalence for the translated items, clarity in language, and cultural appropriateness of the content and methods. See S2 Table for the modified Swahili version of the BDI-11.

### Phase two

#### Aim

To evaluate the latent structure and internal consistency of the BDI-II.

#### Sample

Overall, 221 caregivers were recruited. Of these, 86.7% were female, 9.6% were male while 3.7% had missing data on gender. Moreover, 35.7% of our participants had no formal schooling while 52.9% had primary level education (11.3% missing).

#### Measures

The Swahili version of the BDI-II was administered as an interview by a trained research assistant.

#### Analytic strategy

Confirmatory Factor analysis (CFA) was used to evaluate the factorial structure of the scale. Three factorial models previously reported in the literature are evaluated using CFA. Following suggestions from the literature [17], we assessed the goodness of fit for each model using Chi-square, the Tucker-Lewis Index (TLI), and the Comparative Fit Index (CFI). A non-significant Chi-square is considered ideal, however, since chi square is very sensitive to sample size and model complexity it is often complemented by other fit statistics in evaluating CFA models. Values greater than 0.95 for the TLI and CFI are considered to reflect an excellent fit [17] while those between 0.95 and 0.90 are considered acceptable. The Root Mean Square Error of Approximation (RMSEA) is also reported since it has been shown to be sensitive to model mis-specification: values of less than 0.06 are considered indicative of a good fit [17] while those between 0.06 and 0.08 are considered fair. When one has several competing models to choose from, the model with the lowest Akaike Information Criterion (AIC) is regarded as the most optimal. To evaluate internal consistency we used Cronbach’s alpha. Acceptability of the alpha coefficient is based on those proposed by Cicchetti [18]. To evaluate the relationship between locally derived items and BDI total scores correlational analysis was carried out. Lastly, to investigate the degree of somatization in this population, a repeated measure analysis was carried out.

#### Results

**Fig 1:**
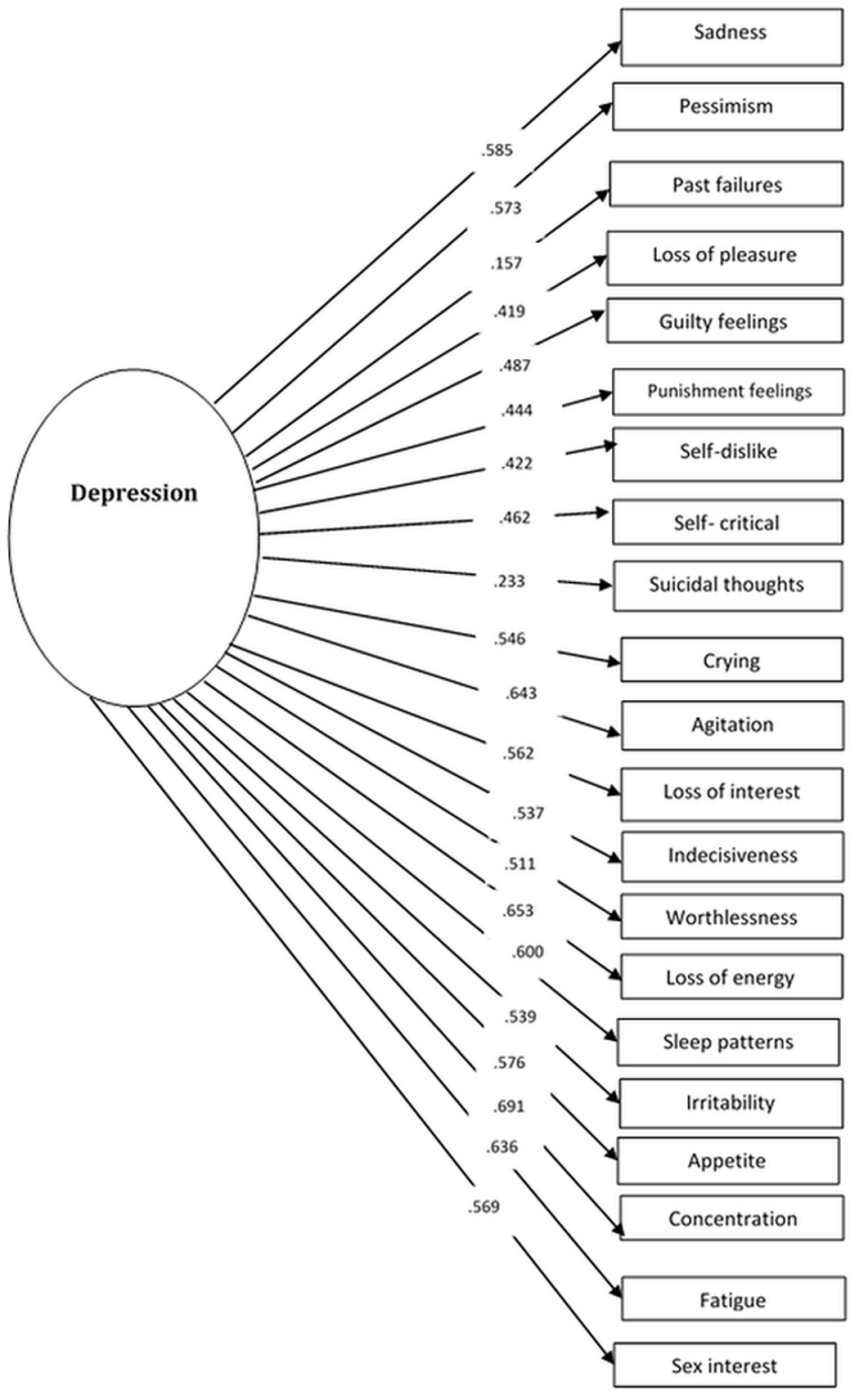
Confirmatory factor analysis (The unidimensional model).

**Fig 2:**
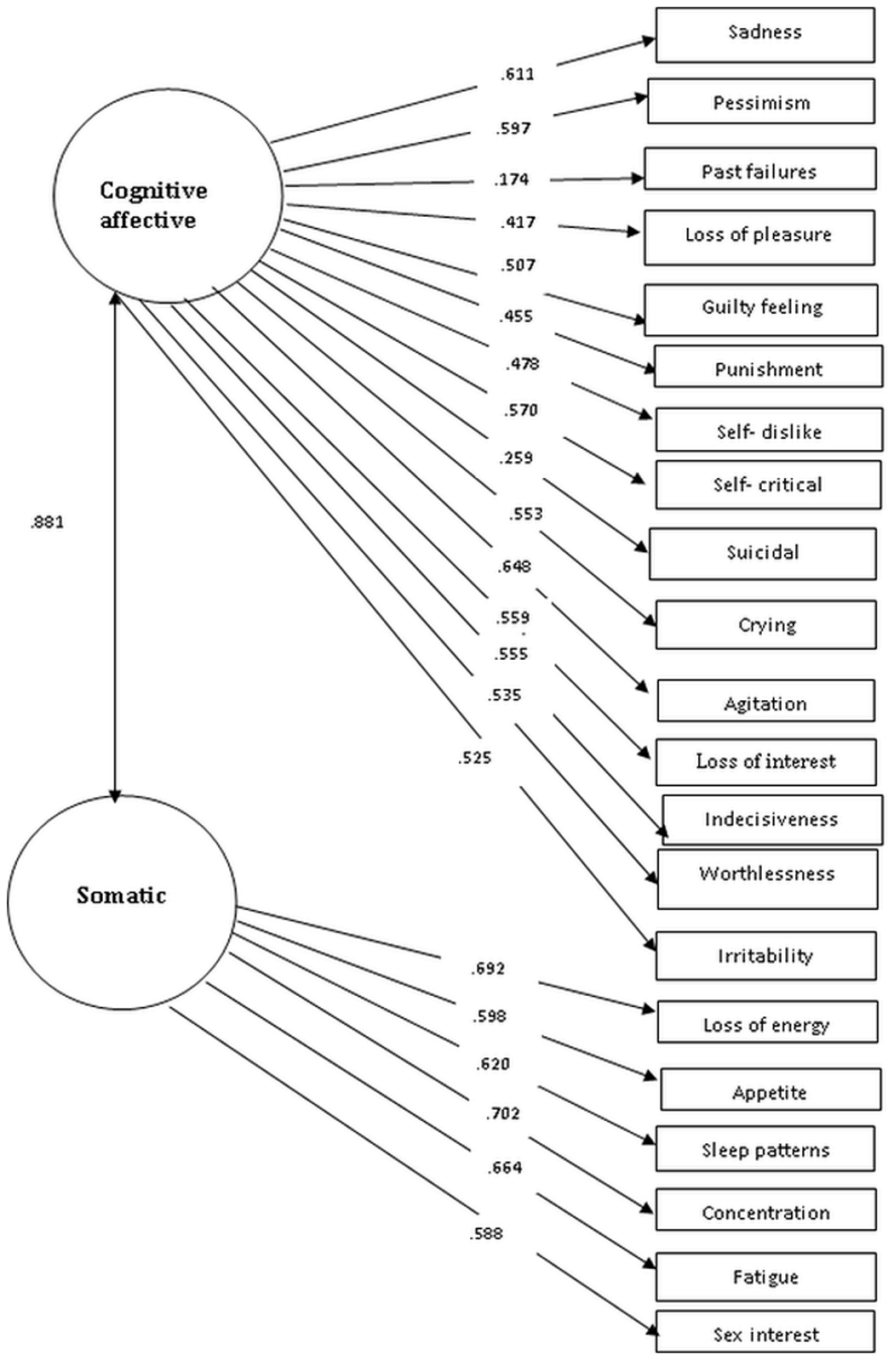
Confirmatory factor analysis (Two factors solution).

**Fig 3:**
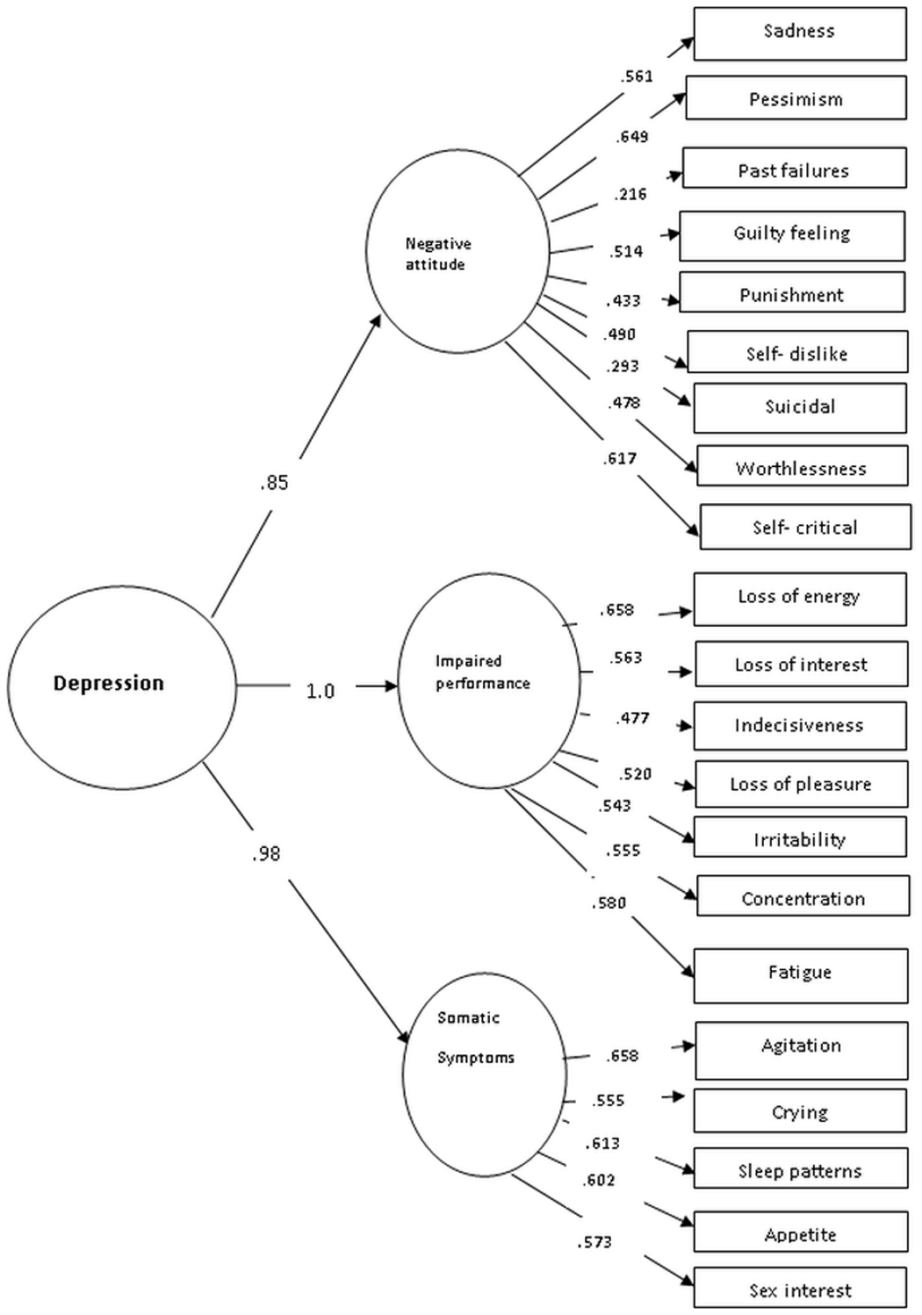
Confirmatory factor analysis (Hierarchical factor solution).

*Confirmatory factor analysis*: Table 1 presents the fit indices of the models assessed, while Figs 1–3 present the tested models and the standardized path coefficients. The one-dimensional model was used as the starting point in our confirmatory factor analysis (CFA); modification indices indicated some errors (items 13, 14 and items 7, 8) that need to be correlated (the decision to correlate the error terms was largely based on the residuals so these two had the largest residual values). Following these modifications this model showed a good fit to the data. We also fitted a two factor model; with one factor evaluating the cognitive-affective aspects of depression and second factor assessing somatic aspects. This model also showed a good fit when some error terms (items 13 & 14) were allowed to correlate. The correlation between these two factors was very strong (*r* = .881). Lastly, we tested a second order three-factor model; this model also showed a good fit to the data.

**Table 1:** Fit Indices for the models.

Model	X^2^	df	CFI	TLI	RMSEA	AIC
One-dimensional model	304.58	187	0.918	0.908	.053	392.58
Two-dimensional model (correlated factors)	300.64	187	0.921	0.911	.053	388.64
Three dimensional model (higher order model)	273.32	186	0.939	0.931	.046	363.32

X^2^ = Chi Square; df = degrees of freedom; CFI = Confirmatory Factor Analysis; TLI = Tucker Lewis Index; RMSEA = Root Mean Square of Approximation and AIC = Akaike Information Criteria

From a statistical point of view, taking into consideration the AIC measure, the best fit to the data was provided by the three-factor model. However, given the strong correlations between the factors, we chose the more parsimonious one-factorial solution. The model retains depression as the overarching, unitary concept. The good fit of the factorial structure of the other models implies that there are symptom groups that show strong within-group relations.

*Internal consistency*: Cronbach’s alpha for the Swahili version of BDI-II was 0.89. Item-total correlations (Table 2, last column) ranged from 0.17 to 0.64; excluding any of the items did not significantly improve the value of alpha. The alpha coefficients and the item-total correlations in this study indicate acceptable levels of internal consistency among the items. In examining the internal consistency of the BDI-II subscales, we found that all the three subscales had acceptable levels of internal consistency: 0.74 for negative attitude, 0.73 for impaired performance) and 0.76 for somatic symptoms. This implies that each of the subscales forms a consistent syndrome thus justifying their use as subscales.

**Table 2:** Item level analyses of the BDI.

Item	Mean	Std. Deviation	Item Total correlation	Mode	Skweness	Kurtosis
Sadness	.89	.86	.556	1	.69	-.21
Pessimism	.81	.85	.556	0	.65	-.61
Past failure	1.10	.74	.171	1	.92	1.21
Loss of pleasure	1.33	.89	.389	2	-.48	-1.18
Guilty feelings	.85	.73	.474	1	.73	.77
Punishment feelings	1.28	1.22	.433	0	.26	-1.52
Self-dislike	.66	.82	.423	0	.84	-.53
Self-critical	.61	.71	.462	0	.95	.47
Suicidal thoughts	.06	.24	.239	0	3.61	11.13
Crying	.66	1.03	.491	0	1.28	.18
Agitation	.86	.88	.595	0	.27	-1.66
Loss of interest	.72	.87	.529	0	.79	-.67
Indecisiveness	.77	.96	.568	0	.93	-.29
Worthlessness	.72	.89	.549	0	1.00	.03
Loss of energy	1.03	.82	.604	1	.30	-.69
Changes in sleep pattern	1.29	1.36	.545	0	.27	-1.77
Irritability	.63	.81	.492	0	.88	-.60
Changes in appetite	.54	.71	.513	0	1.17	.83
Concentration difficulties	.75	.73	.644	1	.49	-.70
Tiredness and Fatigue	.86	.89	.572	0	.39	-1.35
Loss of interest in sex	1.00	1.02	.515	0	.72	-.62

*Correlations with locally identified symptoms*: The three locally added items showed a high level of internal consistency (alpha of .75). Exploratory factor analysis using principal component analysis indicated that these items form a strong single factor (eigenvalue, 2.00), explaining 66.7% of the variance with all items strongly loading on the factor (between .78 and .85). The mean score for the total items was 0.91 (*SD* = 1.11, range: 0, 3). When the scores from these locally identified items were correlated to BDI scores a strong and significant correlation was observed (r [n = 221] = 0.51, p < .001). In examining relationships between the BDI-II subscales and the locally identified items, we found that all the three subscales have a strong and significant correlation with the locally identified item: negative attitude (*r* [n = 221] = 0.40, p < .001), impaired performance (*r* [n = 221] = 0.46, *p* < .001) and somatic symptoms (*r* [n = 221] = 0.48, *p* < .001).

*Syndrome Clusters*: We evaluated the degree to which there were within-person differences in the mean scores of the different syndrome clusters as identified by the second-order three factor model. An inspection of mean scores indicated that the high values were for impaired performance, followed by somatic symptoms and then negative attitudes. A repeated measure analysis indicated that there was a significant difference between the mean scores of negative attitude vs. impaired performance and somatic symptoms but the latter two did not differ (see Table 3). These results indicate that somatic symptoms are not the most salient in this population.

**Table 3:** Means differences between the three subscales of the BDI-II.

(I) Factor 1	(J) Factor1	Mean Difference (I-J)	Standard Error	P value	95% C.I for difference^a^	
					Lower Bound	Upper Bound
Negative attitude	Somatic symptoms	-.093*	.038	.016	-.169	-.018
	Impaired performance	-.094*	.029	.002	-.152	-.036
Somatic symptoms	Negative attitude	.093*	.038	.016	.018	.169
	Impaired performance	-.001	.032	.987	-.064	.063
Impaired performance	Negative attitude	.094*	.029	.002	.036	.152
	Somatic symptoms	.001	.032	.987	-.063	.064

*Content analysis of the open ended question*: Our analysis of the extra information given by participants gave a strong indication of the local idiom to express mental health syndromes. The phrase ‘*kuchoka moyo’* (having a tired heart) or more broadly *‘kuchoka moyo ni kama nina mzigo mzito’* (having a tired heart it is as if you are carrying a heavy burden) seems to come out strongly as a common expression of mental health distress. S3 Table presents a summary of the identified items.

### Phase three

#### Aim

To evaluate the discriminant validity and usefulness of BDI-II in evaluating depressive symptoms in the context of HIV.

#### Sample

The sample involved 106 adults. Of these 29 were community controls (recruited randomly from the community, with an unknown HIV infection status) while 77 were HIV-infected adults recruited from the Comprehensive Care and Research Clinic at Kilifi County Hospital. This clinic specifically focuses on caring for HIV-infected individuals and families. Overall, 80.1% of our adults were females.

#### Measure

The Swahili version of the BDI-II was administered as an interview by a trained research assistant.

#### Analytic strategy

Univariate Analysis of Variance was used to evaluate performance differences between the known HIV-infected and the HIV-uninfected individuals.

#### Results

Our analysis indicated that there was a significant difference between the mean BDI scores of adults randomly recruited from the community (18.20 [*SD* = 8.06]) and scores of caregivers of HIV affected adolescents (24.89 [*SD* = 7.22]) *F*(1, 121) = 23.31, *p* < .001. A noteworthy point is that the mean scores of adults from the community in this study is very close to the mean scores of adults from second phase of the study (18.20 and 17.46, respectively).

## Discussion

The current study set out to adapt the BDI-II into a culturally appropriate and relevant measure of depressive symptoms for use with a low literacy population of Swahili speakers in rural Kenya. Our findings indicate that we were able to make the necessary adaptation yet maintain its factorial structure and reliability.

### Local idioms and symptoms

Both the validity of measures of depression and symptom clustering from Western countries in non-Western countries have created debate. Consequently various earlier researchers have spent a significant amount of effort clarifying the symptom clustering in non-Western settings and validate the instruments. Patel *et al* [7] use notions that are partly ‘emic-based (culture specific or being informed by those from within the culture) and partly etic- based (universal or from outside the culture’ approaches) to develop a culturally appropriate measure. They observed that there was a significant overlap between locally identified items and the items from published measures. This is consistent with this study, in which most of the symptoms mentioned in a free recall exercise overlapped with BDI items. Furthermore, when participants were asked to rate the relevance of the BDI items, no item was rated as irrelevant or difficult to understand.

The locally identified items in our study could easily be clustered into psychological symptoms, physical symptoms and impaired social functioning. Additionally, we observed that there were a few idiomatic expressions and symptoms that featured prominently in the local definition of depression; among them is ‘thinking too much’, ‘having a heavy heart’ or a ‘tired heart’. These idioms refer to a psychological symptom (thinking too much) and a physical metaphor where the heart is very commonly used [19, 20]. Four issues are striking. First, the idioms are very similar to idioms identified in other African settings such as Shona-speakers in Zimbabwe. Second, the potential clustering of these symptoms would closely mirror the findings on the clustering of items on depressive symptoms such as the three factor solution of BDI suggested by Osman *et al*. [21]. Third, even though the word for depression is infrequently used in Swahili and the idiom of distress is not specific for depression but refers to mental distress in general, depression symptoms are strongly clustered, which suggests that depression is well recognized by our informants. From a more practical perspective, given the moderate to high correlations of these terms to the BDI scores, further areas could be explored. Specifically, the correlation between the locally identified items and BDI items indicate that they may have minimal incremental value; however they could be included in a screening tool as a way of increasing ecological validity. Lastly, the identification of various symptoms and idioms that are basically psychological indicates that the earlier findings of the central role of somatisation in defining depressive symptoms in non-Western settings may have been overrated.

A potential administration bias was noteworthy. The four sentence item format was found to be difficult; most of the mothers being interviewed required a lot of repetition to comprehend the options. Furthermore the first person phrasing of the items made the interview difficult. These pattern of results confirmed earlier observations in another rural setting at the Kenya Coast (Mung’ala- Odera, personal communication). The problem with the four item format approach to administering mental health measures has also been previously reported in another low literacy setting [22]. For instance in Pakistan it was observed that the usefulness of the Edinburgh Postnatal Depression Scale (which has a similar format to the BDI) in this context was greatly limited by the difficulty encountered when administering it. Our changes to the administration procedures made it relatively easy to administer the instrument without compromising its factorial structure. This highlights the need for test developers to focus more on developing measures that take into consideration sample characteristics with literacy being an important consideration for resource poor settings. Consistent with what has been observed with earlier studies that adapted psychological measures for rural Kenya [23–25], a systematic approach was successfully implemented in which a Western measure was adapted for use in another cultural context without compromising its psychometric properties. The adapted measure was easy to administer and acceptable to the local community

### Factor structure

In recent years many studies examined the factorial structure of the BDI. This is largely motivated by both the theoretical need to clarify the structure of the BDI (and depression to a large extent), but also by the clinical significance of knowing whether or not a total score or subscale scores need to be used when screening for depressive symptoms. In general the literature presents evidence for various models including two [26–28] and three [29] factors. Our results generally indicate that a single, two- factor and a three factor model all provide an adequate fit to the data. So which is the best way to conceptualization the BDI structure in this setting? In the absence of clear statistical criteria to favour any of these three, we opted for the one-factorial solution as being the most parsimonious. In our dataset it seems that depressive symptoms are best conceptualized as a single unitary factor and this decision is based on using an integration of all existing evidence. Our choice is supported by the good fit of the data on the single model, an excellent alpha for the 21 items, a strong factor loading for almost all the items, and strong correlations between the different factors in the multidimensional models (i.e. the two and three factor models).

The psychometric characteristics of our adapted scale are in line with what has been previously reported. In an extensive review of all earlier reports of the psychometric properties of the BDI [30], it was observed that most studies of BDI-11 report an average alpha coefficient of 0.90 with the value ranging from 0.83 to 0.96, correspondingly closely to the value of 0.89 found in the present study. Moreover, similar to our observation, they report that most studies carried out among a non-clinical sample report item scores on the low end leading to skewness, with suicidal ideation having the lowest endorsement rates.

### Somatisation

In our dataset there was no evidence that somatic symptoms are more common than other symptoms. If this is combined with the high loadings of somatic symptoms, we found clear evidence against a simple somatization hypothesis. Our study indicates that with proper administration procedures and adequate interviewing, members of this community will report psychological symptoms of depression in the same proportion as somatic symptoms.

### Caregivers of HIV affected adolescents

Our results indicate that caregivers of HIV affected adolescents show significantly higher depressive scores compared to a sample randomly selected from the community. These results are consistent with our hypothesis and with earlier published data. For instance a study by Skeen et al [12], indicates that up to 28% of caregivers of children affected by HIV present with common mental disorders when assessed using the Shona Symptoms Questionnaire. The congruence between our results and earlier published works indicates that the adapted BDI-11 is sensitive and can be used to document depressive symptoms among adults living in families affected by HIV.

### Limitations and future directions

We used an interviewer assisted method to collect data to overcome the problems that arise when one is working in a low literate population. However, results arising from this method may be biased due to various self-representation biases during a face-to-face interview, such as social desirability. Indeed a recent report from Zimbabwe indicates that different methods of administering a questionnaire lead to significantly different results [31]. For instance, in the Zimbabwean study, suicidal ideation doubled from 5.3% in interviewer administered questionnaires to 12.0% for computer assisted interviews. The problem of self-representation bias when answering the question on suicidal ideation was likely exacerbated by cultural factors surrounding suicide. In many African communities including the Giryama community where we worked in, suicide is strongly discouraged and stigmatized. So people may feel especially reluctant talking about suicidal ideation. Future research with triangulation may help to gauge method effects [31]. Lastly, our study only confirms the factorial structure of the BDI-II, other important aspects of validity (e.g. Convergent and clinical validity) warrant further research.

### Conclusions

Following a mixed method approach we identified a set of depressive symptoms that closely mirror those of Western countries and observed that the BDI-II maintains a good factorial structure in a rural community following significant adaptations to administration procedures. While new items may be needed to fully cover the spectrum of depression (as found in the interviews) and use of local idioms may help with eliciting the depressive symptoms, an adapted version of the instrument captures many important aspects of depression in this community. We can recommend the use of an adequately adapted BDI-II as part of a package to evaluate mental health outcomes in rural Kenya.

## Supporting Information

S1 Table A summary of the local items identified during the first phase. (DOCX)

S2 Table A Swahili adaptation of the Beck’s Depression Inventory. (DOCX)

S3 Table Extra items mentioned by participants in the open ended questions. (DOC)
