# Supplementary material for: Adaptation and Latent Structure of the Swahili Version of Beck Depression Inventory-II in a Low Literacy Population in the Context of HIV
Source: PLoS One. 2016 Jun 3;11(6):e0151030. doi: 10.1371/journal.pone.0151030 (PMC4892521; doi:10.1371/journal.pone.0151030)
Supplement: S1 Table — (DOCX) [file pone.0151030.s001.docx]

**SI Table 1: A summary of the local items identified during the first phase**

|  | | |
| --- | --- | --- |
| Sad/ unhappy | Not interacting with others | ‘Acting mad’ |
| Restless | Poor job performance | Tired |
| Calm | Poor hygienic standards | Loss of sleep |
| Thinking a lot | May say has been bewitched | Self-blame |
| Irritability | Loses hope in life | Thinks of committing suicide |
| Lonely | Does not sit still | Memory lapses |
| Confused | Not at peace | Weight loss or gain |
| Discouraged | Mixed up thoughts | Think they are worthwhile |
| Complains | Dazed | Talks to themselves |
| Suspicious of other people | Looks worried | Confused |
| Feels isolated |  | Anxious |
