# Supplementary material for: Adaptation and Latent Structure of the Swahili Version of Beck Depression Inventory-II in a Low Literacy Population in the Context of HIV
Source: PLoS One. 2016 Jun 3;11(6):e0151030. doi: 10.1371/journal.pone.0151030 (PMC4892521; doi:10.1371/journal.pone.0151030)
Supplement: S2 Table — (DOCX) [file pone.0151030.s002.docx]

**S1 Table 2: A Swahili adaptation of the Beck’s Depression Inventory**

**Introductory text:** Katika mazungumzo haya ningependa tuongee juu ya hisia zako. Haswa ningependa tuongee juu ya vile umekuwa ukihisi, nguvu zako, kula kwako na mengineyo. Mazungumzo haya ya yanaangalia sana kama kumtokea mabadiliko yoyote katika hisia zako ndani ya wiki hizi mbili zilizopita.

**BDI 1.Katika wiki mbili zilizopita; Je umekuwa na huzuni?**

| **Ndiyo** | **La** |
| --- | --- |
| 1. Unahisi unahuzuni wakati mwingi | 0. Hujihisi unahuzuni |
| 1. Unahisi unahuzuni wakati wote |  |
| 1. Unahisi unahuzuni sana mpaka huwezi kustahimili au kuvumilia |  |

**BDI 2. Katika hizi wiki mbili zilizopita, unapofikiria maisha yako ya usoni/ mbele, je ulihisi umevunjika moyo?**

| **Ndiyo** | **La** |
| --- | --- |
| 1. Unahisi umevunjika moyo kuhusu siku za usoni kuliko ulivyokuwa hapo awali au mwanzoni. | 1. Hujavunijka moyo kuhusu siku za usoni. |
| 1. Hutarajii mambo yako yatafanikiwa siku za usoni |  |
| 1. Unahisi umekata tamaa juu ya siku za usoni na unahisi mambo yatazidi kuwa mabaya. |  |

**BDI 3 :** **Katika hizi wiki mbili zilizopita, unapoangalia maisha yako ya zamani, je unaona umetimiza malengo yako?**

| **Ndiyo** | **La** |
| --- | --- |
| 1. Umeshindwa kutimiza malengo yako kwa kiwango kidogo | 1. Huhisi kama umeshindwa kutimiza malengo yako |
| 1. Unapoangalia nyuma unaona umeshindwa kutimza malengo yako kwa kiwango kikubwa |  |
| 1. Unahisi hujafaulu kabisa maishani |  |

**BDI 4. Kwa kawaida kuna mambo ambayo mtu akifanya humpatia raha. Je katika wiki mbili zilizopita, wewe unahisi vipi unapofanya mambo ambayo kwa kawaida yalikupa raha?**

| **Ndiyo** | **La** |
| --- | --- |
| 1. **Hupati raha kama ulivyokuwa hapo awali** | 1. Bado hupata/huleta raha kama kawaida |
| 1. Wewe hupata raha kidogo kutoka kwenye vitu ambavyo vilikuwa vikikupa raha/furaha. |  |
| 1. Huwezi kupata raha yoyote kutoka kwenye vitu ambavyo ulikuwa ukifurahia/ vikikupa raha |  |

**BDI 5. Katika hizi wiki mbili zilizopita, unapoangalia mambo uliyoyafanya na yale ambayo ulitarajiwa ufanye na hukuyafanya, je unahisi una hatia yoyote ?**

| **Ndiyo** | **La** |
| --- | --- |
| 1. Unahisi una hatia juu ya mambo ambayo umefanya na mambo uliyotarajiwa kufanya na hukuyafanya` | 0.Huhisi kama una hatia yoyote |
| 1. Unahisi una hatia wakati mwingi |  |
| 1. Unahisi una hatia kila wakati |  |

**BDI 6**. **Katika hizi wiki mbili zilizopita, unapoangalia mambo yalivyo maishani mwako, je una hisia zozote juu ya kuadhibiwa?**

| **Ndiyo** | **La** |
| --- | --- |
| 1. Unahisi kama unaweza kuaadhibiwa | 1. Huhisi kama unaadhibiwa |
| 1. Unatarajia kuaadhibiwa |  |
| 1. Unahisi unaadhibiwa |  |

**BDI 7. Kwa kawaida kila mtu anahisia fulani juu ya nafsi yake,baadhi ya watu hujipenda, wengine hujichukia. Je katika hizi wiki mbili zilizopita, kumetokea tofauti yoyote juu ya vile unavyo hisi juu ya nafsi yako?**

| **Ndiyo** | **La** |
| --- | --- |
| 1. Unahisi kukosa kuwa na imani na nafsi yako | 1. Unahisi kawaida juu ya nafsi yako |
| 1. Huridhishwi na nafsi yako |  |
| 1. Hujipendi |  |

**BDI 8**. **Katika ile hali ya maisha ya kawaida watu hujikosoa, Je katika** **hizi wiki mbili zilizopita, wewe umejikosoa zaidi ya kawaida?**

| **Ndiyo** | **La** |
| --- | --- |
| 1. Unajikosoa kuliko ulivyokuwa ukijikosoa hapo awali | 1. Hujikosoi au hujilaumu zaidi ya kawaida |
| 1. Unajikosoa kwa makosa yako yote |  |
| 1. Unajilaumu kwa kila jambo baya linalotokea |  |

**BDI 9. Katika hizi wiki mbili zilizopita, umekuwa na fikira zozote za kujiua?**

| **Ndiyo** | **La** |
| --- | --- |
| 1. Una mawazo/fikira za kujiua lakini hutaweza kuyatimiza | 1. Huna fikira za kujiua |
| 1. Ungependa kujiua |  |
| 1. Ungepata nafasi Ungejiua |  |

**BDI 10. Katika hizi wiki mbili umekuwa ukilia zaidi ya kawaida?**

| **Ndiyo** | **La** |
| --- | --- |
| 1. Unalia zaidi ya kawaida | 1. Hulii zaidi ya kawaida |
| 1. Unalizwa na kila jambo dogo |  |
| 1. Unatamani kulia lakini huwezi |  |

**BDI 11. Wakati mwingine mtu hujihisi kuwa na usumbufu, kutotulia na kuingia kwenye mabishano kwa urahisi, je katika wiki mbili zilizopita umewahi kuwa katika hali kama hii?**

| **Ndiyo** | **La** |
| --- | --- |
| 1. Hujihisi kuwa na usumbufu zaidi ya kawaida | 1. Hakujatokea mabadiliko yoyote |
| 1. Unahisi usumbufu sana au mwenye wasiwasi hata ni vigumu kwako kutulia |  |
| 1. Unahisi usumbufu sana au mwenye wasiwasi hata inabidi uwe katika hali ya kufanya kitu au kutembea |  |

**BDI 12. Katika hizi wiki mbili zilizopita, je kumetokea mabadiliko yoyote katika hamu yako ya kujishughulisha au kujihusisha na watu wengine ?**

| **Ndiyo** | **La** |
| --- | --- |
| 1. Hamu yako ya kujishughulisha au kujihusisha na watu  au mambo imepungua | 1. Hujapoteza hamu yako ya kujishughulisha au kusijihusisha na watu |
| 1. Umepoteza hamu ya kujishughulisha au kujihusisha na watu au mambo kwa kiwango kikubwa |  |
| 1. Ni vigumu kwako kujishughulisha au kujihusisha na jambo lolote/ kitu chochote kile |  |

**BDI 13. Kwa kawaida binadamu hubidi kufanya uamuzi juu ya mambo kadhaa mara kwa mara. Je, katika hizi wiki mbili zilizopita, kumetokea mabadiliko yoyote juu ya uwezo wako wa kufanya uamuzi?**

| **Ndiyo** | **La** |
| --- | --- |
| 1. Unaona ugumu katika kufanya uamuzi zaidi ya kawaida | 1. Unafanya uamuzi wako kama ilivyokuwa awali |
| 1. Una ugumu sana katika kufanya uamuzi kuliko ilivyo kuwa hapo awali |  |
| 1. Una shida kufanya uamuzi wa aina yoyote |  |

**BDI 14. Katika hizi wiki mbili zilizopita kumetokea mabadiliko yoyote ya vile unavyokisia au kukadiria mchango wako katika jamii ?**

| **Ndiyo** | **La** |
| --- | --- |
| 1. Unahisi kuwa wewe huna manufaa/umuhimu/thamani kama hapo wali | 1. Huhisi kuwa huna maanufaa/ au kuwa mimi ni mtu ovyo |
| 1. Unahisi huna thamani au manufaa ukiliganishwa na watu wengine |  |
| 1. Unahisi huna faida/ thamani/manufaa kabisa. |  |

**BDI 15.Katika wiki mbili zilizopita umehisi kuwa na upungufu wa nguvu za kufanya kazi zako za kawaida?**

| **Ndiyo** | **La** |
| --- | --- |
| 1. Nguvu zako zimepungua kidogo | 1. Una nguvu za kawaida |
| 1. Nguvu zako zimepungua sana |  |
| 1. Nguvu zako zimepungua karibu zote |  |

**BDI 16. Katika hizi wiki mbili zilizopita, kumetokea mabadiliko yoyote katika mpangilio wako wa kulala?**

| **Ndiyo** | **La** |
| --- | --- |
| 1a. Unalala zaidi ya kawaida  1b. Unalala kidogo ukilinganisha hapo awali. | 1. Unalala kama kawaida yako |
| 2a. Unalala zaidi sana ya kawaida  2b. Unalala kidogo sana ukilinganisha na hapo awali. |  |
| 3a. Unalala karibu siku nzima  3b. Unaamkaa lisaa limoja hadi mawili mapema kuliko kawaida na huwezi kulala tena |  |

**BDI 17. Katika hizi wiki mbili zilizopita, kumetokea mabadiliko yoyote katika urahisi wako wa kuudhika ?**

| **Ndiyo** | **La** |
| --- | --- |
| 1. Unaudhika kwa urahisi zaidi kawaida | 1. Huudhiki zaidi ya kawaidi |
| 1. Unaudhika mara nyingi zaidi ya kawaida |  |
| 1. Unaudhika wakati wote. |  |

**BDI 18. Katika wiki mbili zilizopita umekuwa na mabadiliko yoyote katika hamu yako ya kula?**

| **Ndiyo** | **La** |
| --- | --- |
| 1a. Hamu yako ya chakula imepungua kidogo sio kama ilivyokuwa hapo awali | 1. Hamu yako ya chakula ni ya kawaida |
| 1b. Hamu yako ya chakula ni zaidi ya kawaida |  |
| 2a. Hamu yako ya chakula imepungua sana sio kama awali |  |
| 2b. Hamu yako ya chakula ni zaidi ya kawaida |  |
| 3a. Huna hamu ya chakula kabisa. |  |
| 3b. Hutamani chakula wakati wote |  |

**BDI 19. Katika wiki mbili zilizopita umekuwa na mabadiliko yoyote katika hali yako ya kutuliza akili yako katika jambo unalifanya?**

| **Ndiyo** | **La** |
| --- | --- |
| 1. Huwezi kutuliza akili yako kwa jambo lolote kama kawaida | 1. Unaweza kutuliza akili yako katika kazi unayofanya kama kawaida |
| 1. Ni vigumu kutuliza akili yako Katika jambo lolote kwa muda mrefu |  |
| 1. Unaona huna kutuliza akili yako katika jambo lolote. |  |

**BDI 20 .Kawaida mtu husikia kuchoka, je katika wiki mbili zilizopita kumetokea mabadiliko yoyote katika kiwango au wepesi wako wa kuchoka?**

| **Ndiyo** | **La** |
| --- | --- |
| 1. Unahisi kuchoka kwa urahisi kuliko kawaida | **0**. Kuchoka au uchovu wako ni wa  kawaida |
| 1. Unahisi kuchoka sana, huwezi kufanya mengi ya vitu ulivyokuwa ukifanya awali |  |
| 1. Unahisi kuchoka huwezi kufanya karibu vitu vyote ulivyokuwa ukifanya hapo awali |  |

**BDI 21.** **Katika hizi wiki mbili zilizopita, je umekuwa na mabadiliko katika hamu yako ya kufanya mapenzi (ngono)**

| **Ndiyo** | **La** |
| --- | --- |
| 1. Hamu yako ya kufanya mapenzi Imepungua kidongo | 1. Hamu yako ya kufanya mapenzi ni ya kawaida |
| 1. Hamu yako ya kufanya Mapenzi imepungua kwa kiwango kikubwa |  |
| 1. Umepoteza hamu ya kufanya mapenzi kabisa. |  |

**Extra Items suggested by the community and local experts:**

**BDI22**: Katika wiki mbili zilizopita umekuwa na fikira nyingi?

0 Hapana 1. Ndiyo

**BDI23**: Katika wiki mbili zilizopita kumekuwa natafouati yoyote ya jinsi unavyojitunza/ au kujiangalia? (yaani kumekuwa natafauti katika unadhifu/usafi wako?)

0 Hapana 1. Ndiyo

**BDI24:** Katika wiki mbili zilizopita umewezakufanya kazi zako ipasovyo? Je, katika wiki hizi mbili umekuwa na matatizo yoyote kufanya kazi vizuri/ kumaliza kazi kwa wakati unaofaa?

0 Hapana 1. Ndiyo

**BDI25:** Je katika wiki mbili zilizopita kumetokea mabadiliko yoyote katika hisia zako, afya yako au maisha yako ambayo mimi sijakuuliza lakini ungependa kunieliezea? **______________________________________________________________________________________________________________________________________________________________________________________________________________________________**

**End time: [____:____] (ETIME)**
