# Supplementary material for: Adaptation and Latent Structure of the Swahili Version of Beck Depression Inventory-II in a Low Literacy Population in the Context of HIV
Source: PLoS One. 2016 Jun 3;11(6):e0151030. doi: 10.1371/journal.pone.0151030 (PMC4892521; doi:10.1371/journal.pone.0151030)
Supplement: S3 Table — (DOC) [file pone.0151030.s003.doc]

**S3 Table: Extra items mentioned by participants in the open ended questions**

|  | **Item** | **Frequency** |
| --- | --- | --- |
| 1 | ‘Tired heart’ some say ’have a tired heart as if I am carrying a burden’ | 26 |
| 2 | Heart beating too fast | 4 |
| 3 | Physical fatigue | 3 |
| 4 | Unhappy | 3 |
| 5 | Mixed up thoughts | 2 |
| 6 | Sleeps a lot | 1 |
| 7 | Easily irritated | 1 |
| 8 | Easily frightened | 1 |
| 9 | ‘Heart jumping/skipping/ shocked | 1 |
| 10 | Worry a lot | 1 |
| 11 | Does not want to interact with people previous close to | 1 |
| 12 | Forgetfulness | 1 |
| 13 | Refusal to speak | 1 |
| 14 | Speaks to one self | 1 |
| 15 | Headaches for no reason | 1 |
| 16 | Speaks to one self | 1 |
| 17 | Headaches for no reason | 1 |
| 18 | Feeling as if your heart has a wound | 1 |
